# A Monitoring and Advisory System for Diabetes Patient Management Using a Rule-Based Method and KNN

**DOI:** 10.3390/s100403934

**Published:** 2010-04-19

**Authors:** Malrey Lee, Thomas M. Gatton, Keun-Kwang Lee

**Affiliations:** 1 Center for Advanced Image and Information Technology, School of Electronics & Information Engineering, ChonBuk National University, 664-14, 1Ga, DeokJin-Dong, JeonJu, ChonBuk, 561-756, Korea; E-Mail: mrlee@chonbuk.ac.kr; 2 The School of Engineering and Technology, National University, 11255 North Torrey Pines Road, La Jolla, CA 92037, USA; E-Mail: tgatton@nu.edu; 3 Department of Beauty Arts, Koguryeo College, Chonnam, Naju City, 520-713, Korea

**Keywords:** diabetes, monitoring system, KNN classifier algorithm, ubiquitous healthcare

## Abstract

Diabetes is difficult to control and it is important to manage the diabetic’s blood sugar level and prevent the associated complications by appropriate diabetic treatment. This paper proposes a system that can provide appropriate management for diabetes patients, according to their blood sugar level. The system is designed to send the information about the blood sugar levels, blood pressure, food consumption, exercise, *etc.*, of diabetes patients, and manage the treatment by recommending and monitoring food consumption, physical activity, insulin dosage, *etc.*, so that the patient can better manage their condition. The system is based on rules and the K Nearest Neighbor (KNN) classifier algorithm, to obtain the optimum treatment recommendation. Also, a monitoring system for diabetes patients is implemented using Web Services and Personal Digital Assistant (PDA) programming.

## 1. Introduction

The use of ubiquitous computing in healthcare service is being actively studied to promote health by implementing systems that will improve the quality of life [1–3]. The number of elderly people and chronic patients in the home is increasing because of the decrease in the birthrate and a demographically aging society that requires increasing management of outpatient health conditions. As a result, the necessity of Ubiquitous Healthcare (U-healthcare) services has increased [1–5].

The goal of ubiquitous computing is to quietly provide services “Anytime, Anywhere, Any Network, Any Device, Any Service,” or “5 Any”, so that the user is not aware of the existence of computers and communication networks providing “Computing, Communication, Connectivity, Contents, Calm,” or “5 C”. For this, a ubiquitous Sensor Network (USN) is needed for connections between electronic spaces and physical spaces, context mining for analyzing user situations and agent analysis techniques to provide the necessary information automatically [6–8].

U-healthcare services include the maintenance and management of health, services supporting healthy maintenance of the body, urgent intervention services for emergencies, services supporting telecommunication and convenient internet access for mobile patients, hospital management services, management services for environmental control, *etc.* An example of a representative U-healthcare service is the Smart Home project known as “Elite Care” which was established in 2000 to accommodate the elderly residents of Milwaukee, Oregon. In this residence, sensors in the Elite Care system alert nurses about the state of elderly people using location tracking badges and indicate any movement outside of a specific region or any abnormal symptoms [9].

There are many cases in which symptoms caused by high glucose levels in the blood do not appear immediately, and if systematic management is not provided, lead to complications such as cataracts, hardening of the arteries, kidney problems, abnormal nervous system conditions, loss of immunity, *etc.* Therefore, diabetes patients must monitor and manage their blood sugar constantly with diet control, exercise therapy, medication, *etc.* However, diabetes patients have difficulty managing this by themselves and they need constant management assistance and the help of their friends and family to maintain a lifestyle in which the diet is coordinated with exercise and activities. Therefore, there is a need for a system that is able to effectively allow control of the blood sugar of diabetes patients.

This paper proposes a system that offers effective treatment advice for diabetes patients, and allows timely management of their blood sugar level. The system is designed to send the information about the blood sugar levels, blood pressure, food consumption, exercise, *etc.*, of diabetes patients, and manage the treatment by recommending and monitoring food consumption, physical activity, insulin dosage, *etc.* The system design is based on rules and the K Nearest Neighbor (KNN) classifier algorithm, to obtain the optimum treatment recommendation. Also, a blood sugar monitoring system for diabetes patients is emulated on a PC and implemented using Web Service and PDA programming in JAVA.

Rule based inference is a method of generalized knowledge representation that deduces the proper results by expressing and selecting the knowledge in a way similar to that of human experts. It is easy to determine the inference based on rules according to the conditions, but such a system is able to make rules only when previous knowledge is available. Therefore, the proposed system integrates the KNN and rule based system approach to generate decisions outside of the strict rules, for diagnosis and the treatment of diabetes. The KNN classifier categorizes results in a structure which represents the results among the classes of other samples of K located most closely with itself in n-dimensional space. The KNN classifier [10–11] is the simplest machine learning algorithm for calculation of the Euclidean distance. It is able to classify results using given sample data without previous knowledge, and if the number of dimensions is small, it is appropriate for small-sized data. Therefore, the system is designed to select the method of treatment using the KNN classifier to evaluate time, blood sugar, blood pressure, number of meals, amount of exercise and target caloric consumption.

Section 2 next introduces the concept of U-healthcare as applied to diabetes in related studies and case studies. Section 3 presents an outline of a blood sugar monitoring system, a treatment management system and a calorie calculator. It introduces the treatment decision mechanism and the associated rule based and KNN classification and implementation methods. The experimental performance is presented in Section 4. Finally, Section 5 describes the results of this research and the recommendations for future research and study.

## 2. Applications for Diabetes in U-Healthcare

Georgetown University developed the “MyCare System” [12] which is a two-way Internet-based diabetes management system. This system transmits the patient’s blood sugar level, which is measured by a blood sugar tester, to a database. Then, the patients and doctors can access the related data through Internet browsers. Also, by allowing the blood sugar information to be analyzed automatically for patients and doctors, it helps doctors make decisions about the recommendations for meals, exercise and medication. This approach helps patients to manage their disease more actively and to have a better understanding of their disease.

Glocowatch [13–14] was developed by the Cygnus Company using a non-invasive testing method that uses biosensors, which are the most convenient form of blood sugar sensors developed to-date for diabetes patients. There are many advantages of this sensor, but people who have sensitive skin sometimes develop side effects when they are swimming or bathing. Also, there have been issues regarding its accuracy when the skin conductance changes, for example when the patient sweats. This method is not recommended for patients with this problem and traditional blood-gathering methods must be used.

In Denmark, Aarhus University has a project entitled “Tele-medical examination of Diabetic Patients with Foot Ulcers”, which uses technology based on Ubiquitous IT. The “Tele-medical examination of Diabetic Patients with Food Ulcers” system is composed of scenarios. There are many cases with patients who have suffered from diabetes for a long time and whose blood supply has decreased and caused nerve destruction. The system allows home nurses to visit the patient’s home, check the state of their legs and send the related image information to the doctors. Then, the doctors check the state of the patient’s leg through the image information and issue a prescription for the patient. Through these processes, those patients who have serious leg pain can reduce their visits to the hospital. Also, the examination time required for doctors can be reduced, because the home-nurses and doctors discuss the state of the patient before prescribing the treatment. This system is able to provide more suitable medical services to patients by sharing the patient’s information with home-nurses and doctors [15].

In South Korea, the “Diabetes phone” was developed jointly by Healthpia and LG Electronics [16]. The “Diabetes phone” can also directly measure the blood sugar level, send it to the doctors and save it. It also can provide services such as exercise control, meal therapy, medication control, diabetes education, *etc.*, based on that information. It also allows the amount of exercise per day to be measured. To obtain blood sugar level information, litmus paper is used to gather blood from the fingertip and then the patient touches a recognition system that is built into the cellular phone. In this way, the patients can measure their blood sugar and immediately find out how much blood sugar is in their blood [9,17–18]. Japan developed the “Home diabetes patients supporting system”. It aims to improve the control of blood sugar levels and prevent complications by utilizing self-measured blood sugar information obtained using the mobile internet. In order to utilize the self-tested blood sugar information obtained every day in the home, while traveling, *etc.*, by the home diabetes patients, it registers the data in the database server at Tougane hospital using information instruments such as the mobile internet, a PC, or a Fax. In the case of the mobile internet, it signals the blood sugar tester and conducts an automatic transfer of the self measured blood sugar information. The blood sugar data is encrypted for transmission. After sending the self measured blood sugar information, it analyzes the measured data in the Home diabetes patients supporting system, and the analyzed results and doctor recommendations are sent to the patient’s reception device [5].

In the case where biosensors are used, the accuracy decreases, however it is more convenient and lowers the pain experienced by patients, because there is no need to obtain blood samples. Also, cases where nurses visit the home, check the patients and receive the tele-prescriptions from doctors require a lot of time and manpower, which these systems eliminate. The Diabetes phone and Japan's Home diabetes patient support system use a blood sugar tester and record the blood sugar information and the prescription given by the doctors. Currently, only the blood sugar is managed by the system, and if the doctor leaves the hospital, it is difficult to provide advice and consultation. Currently, the website of Healthpia is not active [19].

This paper describes the implementation of a system that transmits the proper treatment method to the patients using their PDA. In order to avoid inconveniences, the information is obtained from a blood sugar tester, blood pressure tester, and a treadmill using Radio Frequency Identification (RFID). The system informs the patient of the recommended treatment method and compares it with the method based on rules the KNN classifier. The system is emulated on a PC and implemented using a Web Service and PDA programming in JAVA, using the method which provides the best result between the rule-based and KNN classifier methods.

## 3. Learning Method of a Blood Sugar Monitoring System

This Section introduces the blood sugar monitoring agent system and presents the approach used to determine the proper treatment recommendation. This approach uses a decision method based on rules and the KNN classifier.

### 3.1. Outline of the System

**Figure 1.**
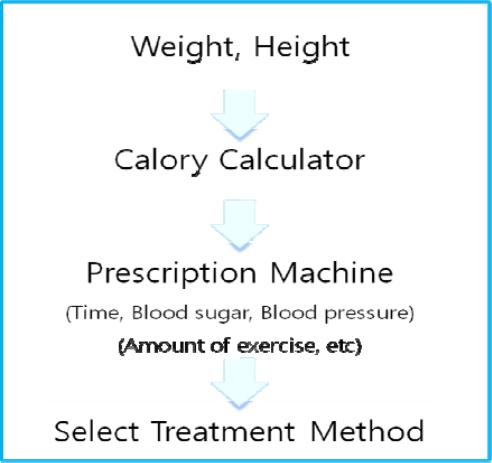
Diagram of the blood sugar monitoring agent system.

The blood sugar monitoring agent system for diabetes patients manages the meal and exercise therapy plans with the process shown in Figure 1, and is based on the information obtained from the RFIDs.

The diabetes patients must maintain a reasonable weight by proper diet and exercise in order to control their blood sugar and prevent diabetic complications. The blood sugar monitoring agent system for diabetes patients calculates the number of calories which the patient should consume per day, according to the patient’s body information, and manages the meal and exercise therapy for proper blood sugar levels and diabetes treatment optimization [5,20,22]. The patient’s name and age are obtained through direct input by the patients, and related health information, such as blood sugar, blood pressure, weight, *etc.*, is obtained using RFIDs and an attached tester. The information sent from the tester assumes that the information is textual and is input as text. This experiment deals with the patient’s state, which consists of the daily caloric consumption, blood sugar, meals, and exercise levels, which are measured by the blood sugar monitoring agent system.

Table 1 shows the number of calories which patients must consume per day [20]. For example, a woman whose height is 165 cm, or 1.65 m, has a standard weight calculated by the formula:

1.65 m×1.6 m×21=57.1725 kg

Thus, the standard weight of the woman is about 58kg, and she belongs to the light activity type, so the number calories which she must consume per day is calculated using the formula: standard weight (kg) × 25 (kcal/day). Because her standard weight is 58 kg, the number of calories which she must consume per day is 58 kg × 25 kcal/day = 1,450 kcal.

After calculating the number of calories which the patient should consume per day, the measured data, which includes measurement time, blood sugar, blood pressure, amount of exercise, *etc.*, is acquired from the RFIDs, and the user inputs the food consumption data. Then, the software chooses the optimum treatment method, after comparing the rule and KNN classifier recommendations.

### 3.2. The Treatment Method Decision

**Figure 2.**
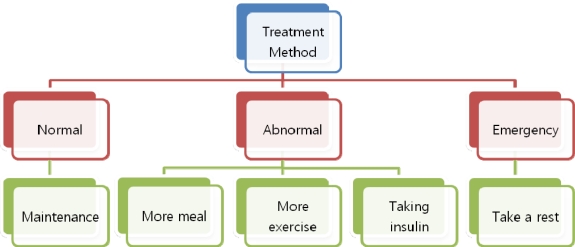
The type of treatment method.

There are 5 types of treatment methods considered in the proposed system, as shown in Figure 2. First, the patient’s current condition is categorized as normal, abnormal or emergency. Patients in a normal state simply maintain that state. When the patient’s state is abnormal, they can eat more, exercise more, or take insulin. In an emergency situation, the patient should rest.

In the program architecture and construction, this paper compares the method based on rules with the method using the KNN classifier. The system rules are built by using diagnostic knowledge about diabetes and its associated treatment methods, and the KNN classifier is established by selecting suitable diagnosis from 300 sets of experimental data.

### 3.3. The Method Based on Rules

Rule-based inference has been used for a long time and is a method for generalized knowledge representation. Rule-based inference deduces the proper results by expressing and selecting the knowledge in a way similar to human experts [20].

The condition portion of the rules is shown and the decision is the logical product or the logical sum of the condition. If the condition portion of the rule is true, its conclusion is set to true [9]. The inference based on rules is easy to determine according to the conditions, but has the disadvantage that the rules can only be established from previous knowledge.

The diagnosis of diabetes is made as shown in Table 2. This diagnosis assumes that the subject’s meal times are 7:00, 12:00 and 18:00, and that their sleeping time is 24:00. The treatment method is determined by distinguishing between an empty and full stomach, as shown in Table 3.

### 3.4. The Method Using the KNN (K Nearest Neighbor) Classifier

KNN [21] is the most convenient machine learning algorithm not requiring prior learning. There are advantages when using sample data and it is suitable for small scale data. The KNN classifier is one of the non-parametric learning methods which are not affected by the distribution state of the sample. Samples exist as n-dimensional space points and the vector of sample x is expressed as:

$$<a_{1}(x),a_{x}(x),\ldots ,a_{n}(x)>$$

The distance between the two samples, (*X_i_* and *X_j_*), is *d* (*X_i_*, *X_j_*) and can be determined assuming that expression 1 corresponds to the Euclidean distance.

(1) $$d(x_{i},x_{j})=\sqrt{\sum_{r=1}^{n}{\left(a_{r}(x_{i})-a_{r}(x_{j})\right)}^{2}}$$

If the target function having discrete-values is *f : R^n^* → *V*,*V* = {*v*_1_,*v*_2_,...,*v_n_*}, the class of *x_q_* and *f̅*(*x_q_*) can be described by expression 2:

(2) $$\begin{array}{c} \bar{f}(x_{q})\leftarrow \text{arg}\;\text{max}\sum_{i=1}^{k}\delta (v,f(x_{i}))\\ v\in V\\ \left(\text{if}\;a=b,\;\text{then}\;\delta \left(a,b\right)=1,\;\;\;\text{otherwise}\;\delta \left(a,b\right)=0\right) \end{array}$$

This is the KNN algorithm that classifies many things among the k of other sample classes most closely located in n-dimensional space [21]. The KNN classifier is trained with 200 sample data sets and tested on 100 test data sets. The structure of the two data sets consists of the sample time, blood sugar, systolic blood pressure, diastolic blood pressure, amount of exercise, meal consumption, target consumption calories, and the treatment method.

The expression used is the same as the formula used to calculate the Euclid distance using the KNN, as shown in expression 3:

(3) $$d(x_{i},x_{j})=\sqrt{\sum_{r=1}^{n}{\left(a_{r}(x_{i})-a_{r}(x_{j})\right)}^{2}}$$

It calculates using the formula shown in expression 4 using sample and test data where *t1* is the test data, *t2* is the sample data, time is *t*, blood sugar is *bs*, systolic blood pressure is *bh*, diastolic blood pressure is *bl*, the amount of exercise is *ce*, the meals are *cm*, and the target consumption calories is *c*:

(4) $$d(t1,t2)=\sqrt{\begin{array}{ll} {\left(t_{t1}-t_{t2}\right)}^{2} & +{\left({\mathrm{bs}}_{t1}-{\mathrm{bs}}_{t2}\right)}^{2}+{\left({\mathrm{bh}}_{t1}-{\mathrm{bh}}_{t2}\right)}^{2}+{\left({\mathrm{bl}}_{t1}-{\mathrm{bl}}_{t2}\right)}^{2}\\ & +{\left({\mathrm{ce}}_{t1}-{\mathrm{ce}}_{t2}\right)}^{2}+{\left({\mathrm{cm}}_{t1}-{\mathrm{cm}}_{t2}\right)}^{2}+{\left(c_{t1}-c_{t2}\right)}^{2} \end{array}}$$

The program selects the smallest number and compares the treatment method of the sample data and the treatment method corresponding to the data. The small scale data that is used consists of the KNN classifier and the time, blood sugar, systolic blood pressure, diastolic blood pressure, amount of exercise, amount of meals, and target consumption calories. It is used because it is the simplest machine learning algorithm, and is able to classify data without any human expert assistance.

When K = 1, the algorithm selects the sample data set having the lowest calculated value in that data set. If K = 3, it selects three sample data sets with the lowest calculated value out of one test data set. It selects the most appropriate treatment method among the three methods of treatment according to the appropriate principle of majority rules. When K = 5, it selects five of the sample data having the lowest calculated value from one set of test data. It selects the most appropriate treatment method among the five methods of treatment according to the appropriate principle of majority rules. This paper compares the cases where K = 1, K = 3, and K = 5. Also, if the gap between each set of data becomes large, the calculated value of the KNN classifier becomes large and does not produce good results. Therefore, it reduces the gap between each data set through normalization and compares it with cases where the data is not normalized.

## 4. Experimental Evaluation

This Section introduces the data used in the experiment and compares the rule based method with the method that uses the KNN classifier to select the most appropriate treatment method. Also, it describes the implementation of a blood sugar monitoring system designed to select the best treatment method through the process of comparison and using the patient’s PDA. To implement the proposed system, a mobile RFID module is designed and developed. A Wireless Internet Platform for Interoperability (WIPI) interface is developed as an emulator application for the mobile environment in JAVA using a Pico pass/Pico RFID tag and a HAND' IT-2G RFID reader. Table 4 shows a portion of the sample data and the test data. Time has values ranging from 0 to 24, the blood sugar and blood pressure are measured data. The units for exercise, amount of meals and target caloric consumption is in kcal. The amount of exercise is the number calories consumed after exercise, the amount of meals is the number of calories consumed after a meal, and the target caloric consumption is the total number of calories that the patient must consume per day. The treatment is one of the five methods of treatment as listed in Table 5.

### 4.1. The Method Based on Rules

**Figure 3.**
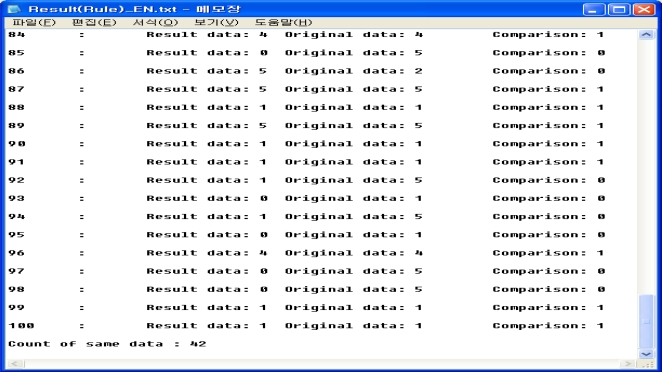
The results of the rule-based method.

**Figure 4.**
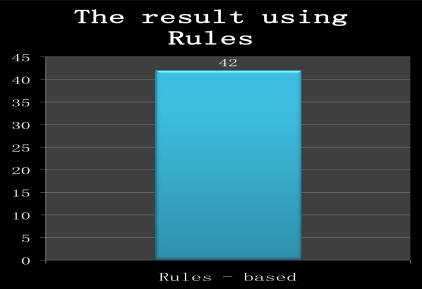
The results of the rule-based method.

The test data is processed and the resulting values comprise the treatment recommendations of the blood sugar monitoring system based on rules. Samples of the experimental rule-based results are shown in Figure 3. The resulting values are the treatment method that is selected from rule-based inferences and the numerical value is the treatment method identified from the test data. If two values are the same, then set is 1. Otherwise it is set to 0. The match of 42 among 100 total test data is shown in Figure 4.

### 4.2. The Method Using the KNN Classifier

#### 4.2.1. The non-normalized data

**Figure 5.**
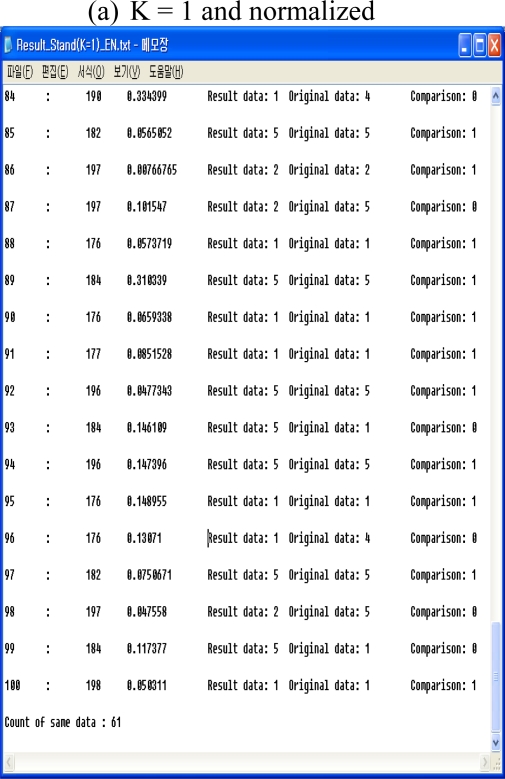

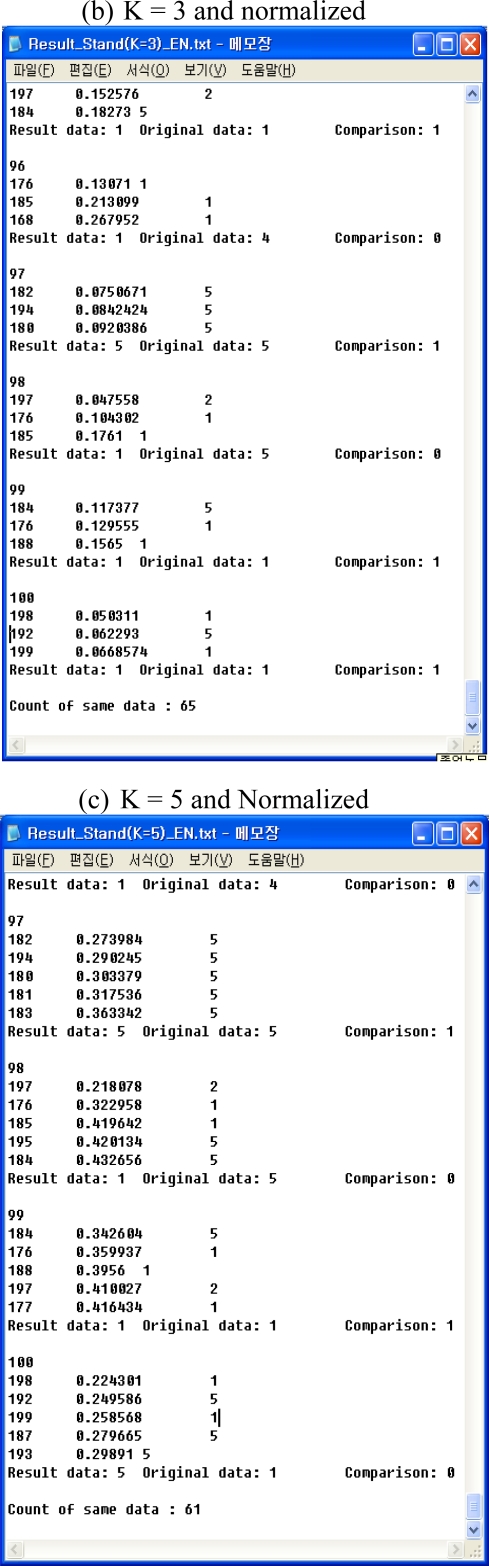
The result of KNN normalized.

The results are calculated from a sample data set and are in contrast to the test data, having three related when K = 1, K = 3, and K = 5. Figure 5 shows the results when K = 1, K = 3, and K = 5. If the treatment values that were selected as the result values have the same treatment values as the original test data, it is set to 1, and otherwise it is set to 0. It is the same for 36 when K = 1, and the same for 52 when K = 3, and the same for 64 when K = 5 for the 100 data sets.

#### 4.2.2. The normalized data

The sum of the squares of each difference becomes comparatively large if the difference in size between the data becomes large and it is not possible to obtain good results. To address this problem, the data is normalized using the formula in expression 5:

(5) $$\mathrm{Normalized}\;\mathrm{data}=\frac{\mathrm{Current}\;\mathrm{Value}-\mathrm{Minimum}}{\mathrm{Maximum}-\mathrm{Minimum}}$$

The data are normalized so that their maximum value is 1 and their minimum value is 0, using the above formula. The maximum is the maximum value of the sample data and the minimum is the minimum value of the sample data. The current value is the input value of the test data. If the normalized data is larger than 1, it is set to 1. If the normalized data is smaller than 0, it is set to 0. Because the goal of the treatment selection is to obtain the best of five treatment methods, the treatment is not normalized, as shown by the normalized sample data and test data in Table 6.

The calculations are obtained for K = 1, K = 3, and K = 5. Figure 5 shows the results for K = 1, K = 3, and the normalized results for K = 5.

**Figure 6.**
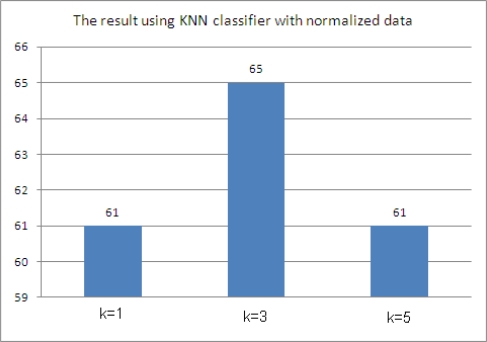
Results using the KNN classifier with the normalized data.

If the selected treatment value is the same as the original test value, the result is 1. Otherwise it is 0. The results when K = 1 are that 61 out of 100 are the same, 65 are same when K=3, and 61 are the same when K = 5, as shown in Figure 6.

### 4.4. Comparison

**Figure 7.**
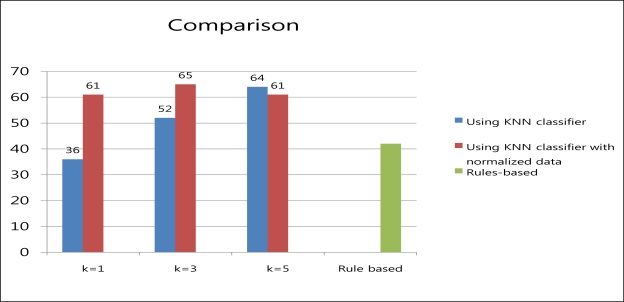
Comparison.

Both the blood sugar monitoring agent systems using the KNN classifier and the KNN classifier with the normalized data obtained the best results when K = 3. Also, the blood sugar monitoring agent system based on rules obtained similar results to the blood sugar monitoring agent system using the KNN classifier. However, the rule based method based requires human expert input, because formulating the rules requires previous knowledge about diabetes. The blood sugar monitoring agent system using the KNN classifier with the data normalized between 0 and 1 obtained the best results among the three methods. Therefore, the blood sugar monitoring agent system using the KNN classifier with normalized data, to reduce the gaps between the data, identifies the most appropriate treatment method. Also, the KNN algorithm is able to classify data easily using test data without prior learning. Thus, it can select the method of treatment without the help of a human expert possessing medical knowledge of diabetes. Figure 7 shows a comparison of the results.

### 4.5. Implementation

In this experiment, the method using the KNN classifier with normalized data when K = 3 obtained the best result between the method based on rules, the method using the KNN classifier, and the method using the KNN classifier with normalized data. Therefore, the blood sugar monitoring agent system for patients using a PDA was implemented utilizing the method based on the KNN classifier with normalized data when K = 3. If all of the calculations are performed on the PDA, the system is inefficient, with the result that the speed of the system is slow and all of the information must be saved on the server. Therefore, the Web Service, ‘BloodSugarService’ is implemented on the server to transfer the calculated result to the PDA through communication between the PDA and the server. The Web-Reference of the “BloodSugarService.” Web Service for the program shows a PDA Web Service that has a list of tasks, such as the calculation of the number of calories, the transfer of the information and the selection of the treatment method.

**Figure 8.**
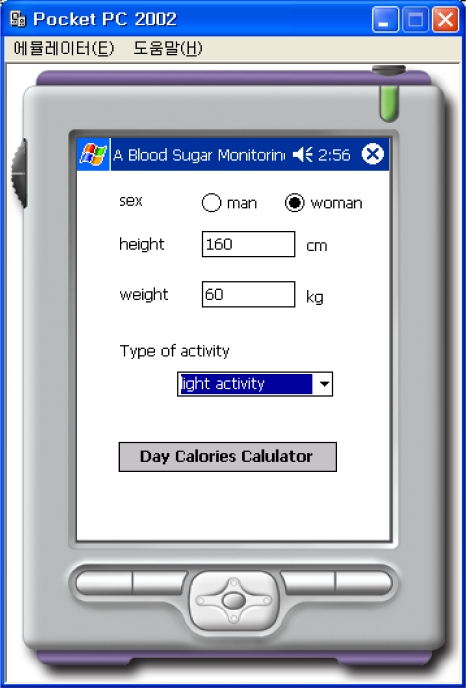
Input values for Daily Calories.

**Figure 9.**
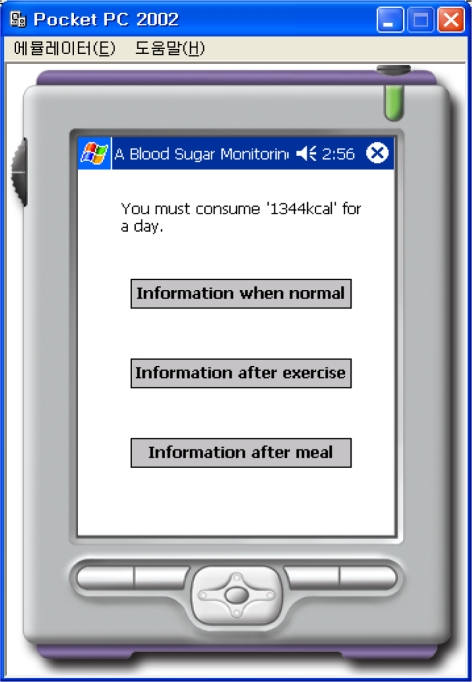
Threes Types of Information.

**Figure 10.**
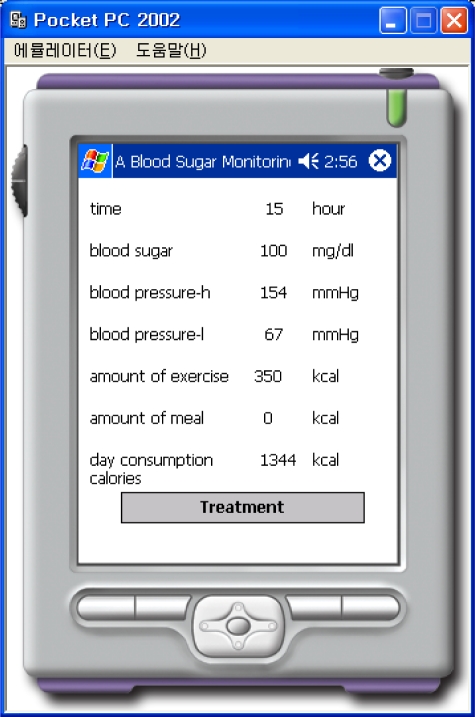
Result for Normal.

This “BloodSugarService” Web Service uses the web-reference in order to do the PDA programming. Information such as the sex, height, weight, and types of activities are input, as shown in Figure 8 in order to calculate the number of calories that must be consumed per day. The calculation is performed by connecting to the “BloodSugarService” Web Service, and information about the number of calories that must be consumed per day is displayed on the PDA. As shown in Figure 9 there are three buttons labeled normal, after exercise, and after a meal. The information about time, blood sugar, systolic blood pressure, diastolic blood pressure, amount of exercise, amount of meals, calories consumed per day, *etc*., may be retrieved from the file and displayed on the screen, as shown in Figure 10, when the patient presses the appropriate button.

**Figure 11.**
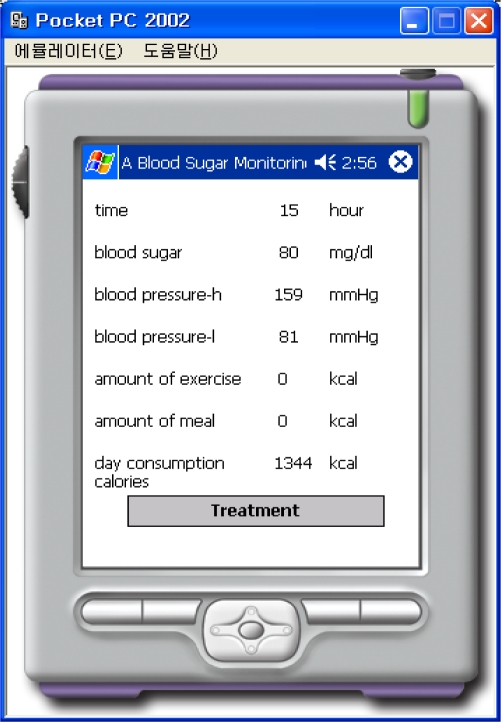
Result of After Exercise.

**Figure 12.**
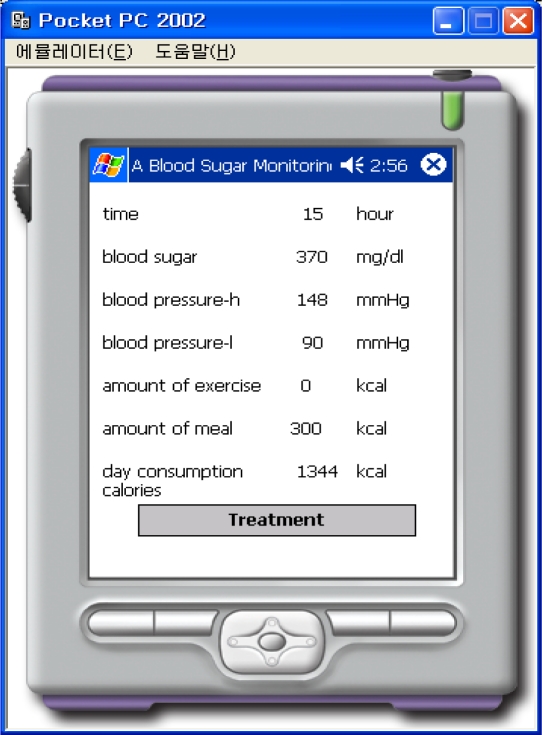
Result of After Meal.

Information for the normal case, where the amount of exercise and the number of meals is 0, is shown in Figure 11. Figure 11 corresponds to the case after exercise and Figure 12 corresponds to the case after a meal.

**Figure 13.**
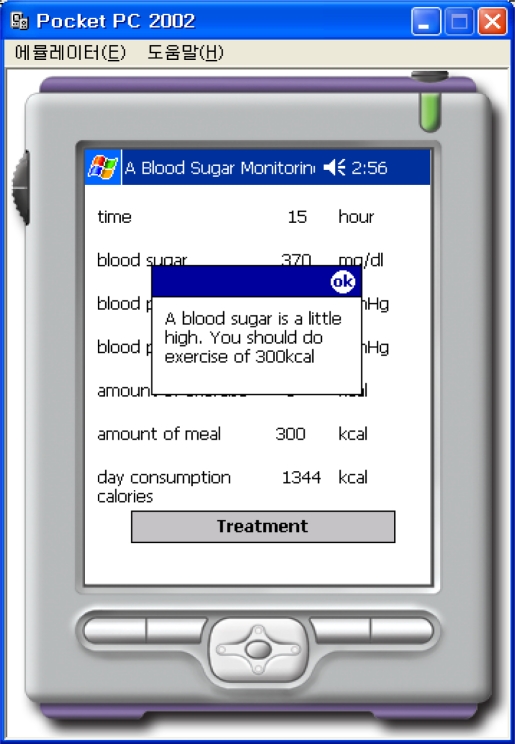
Result of Treatment.

If the patient clicks on the prescription button, the system connects to the “BloodSugarService” Web service that uses the normalized data as input to the KNN classifier for K = 3. The calculation is performed with the sample data on the server computer using the information such as the time, blood sugar, systolic blood pressure, diastolic blood pressure, amount of exercise, amount of meal, calories, *etc.* The PDA is informed of the results in the form of a prescription, as shown in Figure 13 based on the calculated results. According to the prescription, the patient should eat more, do more exercise, take insulin, take a rest, or continue as normal.

## 5. Conclusions

In this paper, a blood sugar monitoring system for diabetes patients was described where information, such as the blood sugar, blood pressure of the diabetes patient, amount of meals, amount of exercise, *etc.*, is acquired using RFIDs. Based on this information, a software program was implemented in JAVA and the system was able to recommend appropriate treatment by advising the patient about meal and exercise control, taking insulin, *etc.* It calculates the number of calories per day that the patient must consume and manages their meal and exercise therapy, along with blood sugar level, in accordance with the patient’s body condition. The patient monitored information is sent from the RFIDs as a text file. If the blood sugar is high, the patient should exercise in order to lower their blood sugar. If the blood sugar is low, the patient should eat more in order to increase their blood sugar. In the case where the blood sugar is consistently too high and exercise therapy is insufficient, the treatment method prescribed is “Taking Insulin”. The prescription treatment algorithm was designed and the method based on rules was compared to the method using the KNN classifier, and the optimum treatment method was identified.

The method based on rules needs prior information about diabetes. Therefore, the prescription algorithm was designed by using rules for the diagnosis and treatment of diabetes, to select the optimum treatment method based on rules. The prescription treatment algorithm was designed to use the KNN classifier. The prescription algorithm method based on rules has the disadvantage that is not able to select any treatment method without previous knowledge about diabetes. However, the prescription algorithm using the method based on the KNN selects the optimum treatment method simply by using the given sample data. The KNN classifier was calculated by inputting the seven factors of time, blood sugar, systolic blood pressure, diastolic blood pressure, amount of exercise, amount of meals and target consumption calories. It was able to select the most optimum treatment method without difficulty, because the dimensions are comparatively small. After comparing the two methods, the prescription algorithm using the KNN classifier was selected as the optimum treatment method, rather than the prescription algorithm based on rules. The normalized data input into the KNN classifier was compared to non-normalized data, to identify the approach that provided better results. The method using the KNN classifier was found to yield better results and a blood sugar monitoring agent system for patients is emulated on a PC using the Web Service and PDA programming in JAVA was implemented that used the patient’s PDA and the KNN classifier to normalize the data.

This study implemented a system that can be applied to diabetes only. Therefore, more research is needed to develop a system that can be applied to chronic diseases which can be caused by diabetes, such as high blood pressure, hardening of the arteries, cataracts, nephropathy, *etc.* Specific patient information must be represented and implementation extension for this feature is straightforward. The primary purpose of the research is to demonstrate feasibility of the approach, and representation of all possible cases for insulin recommendations is recommended for future work. Further study is also needed to identify other algorithms for application, such as a neural networks, genetic algorithms, *etc.* Finally, it is proposed that it is possible to more correctly and appropriately treat diseases in real-time using RFID technology that allow capturing and transmission of a patient’s current condition.

## Figures and Tables

**Table 1.** The day consumption calories by activity.

**Type of activity**	**Example of activity**	**The day consumption calories**

light activity	sedentary work, drive, office work, *etc.*	standard weight (kg) × 25∼30 (kcal/day)
normal activity	walking, cleaning, light industries, housework, *etc.*	standard weight (kg) × 30∼35 (kcal/day)
hard activity	mountain-climbing, transport heavy load, extreme exercise, *etc.*	standard weight (kg) × 35∼40 (kcal/day)

**Table 2.** The standard diabetes diagnosis [16].

**Normal fasting plasma glucose (FPG) level**	**Blood sugar after 2 hours of meal ingestion**	**Results**

below 100 mg/dL	below 140 mg/dL	normal
over 126 mg/dL	over 200 mg/dL	abnormally high glucose levels
below 100 mg/dL	140 mg/dL–199 mg/dL	impaired glucose tolerance (IGT)
100 mg/dL–125 mg/dL	below 140 mg/dL	impaired fasting glucose (IFG)
below 70 mg/dL	-	low blood sugar

**Table 3.** To select rules of diabetes treatment method.

**Division**	**Time(hour)**	**Treatment**

Normal fasting plasma glucose (FPG) level	0–7 9–12 14–18 20–23	If the blood sugar is over 126 mg/dL, then do more exercise.
Before sleeping	22–24	If the blood sugar is below 120 mg/dL, then eat more.
4 hours after meals	9–12 15–17 20–23	If the blood sugar is over 140 mg/dL, then do more exercise; if it is below 100, then eat more
Always	0–24	If the blood sugar is below 70 mg/dL, then rest; If it is over 180 mg/dL, then take insulin.

**Table 4.** Experimental data.

**Time (hour)**	**Blood sugar (mg/dL)**	**Systolic blood pressure (mmHg)**	**Diastolic blood pressure (mmHg)**	**Amount of exercise (kcal)**	**Amount of meals (kcal)**	**Target caloric consumption (kcal)**	**Treatment**

5	84	140	69	240	0	1,267	5
7	199	136	65	0	200	1,344	1
11	75	161	75	0	0	1,653	2
19	300	164	68	0	0	600	4
7	50	140	65	300	0	1,344	3
8	134	143	63	350	0	2,000	1
7	57	136	64	0	370	2,439	3
23	100	120	84	0	0	150	2
13	196	133	83	0	351	2,000	1
12	102	152	76	0	340	2,000	5

**Table 5.** The treatment method.

**Expression**	**The treatment method**

1	more exercise
2	eat more such as a meal or snack
3	emergency (rest)
4	take insulin
5	normal (maintenance)

**Table 6.** The normalized data.

**Time**	**Blood sugar (mg/dL)**	**Systolic blood pressure (mmHg)**	**Diastolic blood pressure (mmHg)**	**Amount of exercise (kcal)**	**Amount of meal (kcal)**	**Target consumption calories (kcal)**	**Treatment**

0	0.0199	0.5	0.38	0.48	0	0.752	5
0.111	0.399	0.45	0.3	0	0.294	0.798	1
0.333	0.067	0.7625	0.5	0	0	0.984	2
0.777	0.670	0.8	0.36	0	0	0.35	4
0.111	0	0.5	0.3	0.6	0	0.798	3
0.167	0.225	0.5375	0.26	0.7	0	1	1
0.111	0.0188	0.45	0.28	0	0.544	1	3
1	0.134	0.25	0.68	0	0	0.081	2
0.444	0.391	0.4125	0.66	0	0.516	1	1
0.389	0.139	0.65	0.52	0	0.5	1	5
